# Retinopathy of Prematurity: Incidence, Risk Factors, and Treatment Outcomes in a Tertiary Care Center

**DOI:** 10.3390/jcm13226926

**Published:** 2024-11-17

**Authors:** Mara Nike Blazon, Sandra Rezar-Dreindl, Lorenz Wassermann, Thomas Neumayer, Angelika Berger, Eva Stifter

**Affiliations:** 1 Department of Ophthalmology and Optometry, Medical University of Vienna, Waehringer Gürtel 18-20, 1090 Vienna, Austria; 2 Department of Pediatrics and Adolescent Medicine, Division of Neonatology, Intensive Care Medicine and Neuropediatrics, Comprehensive Center of Pediatrics, Medical University of Vienna, Waehringer Gürtel 18-20, 1090 Vienna, Austria

**Keywords:** preterm infant, retinopathy of prematurity, anti-VEGF, risk factors, epidemiology, laser coagulation

## Abstract

Retinopathy of prematurity (ROP) remains a major cause of childhood blindness. Its pooled prevalence worldwide is 31.9%, and that of severe ROP is 7.5% among prematurely born babies. Investigating risk factors is essential for improving early detection and treatment outcomes. **Purpose**: To determine the frequency and stages of ROP cases and evaluate the treatment methods for premature infants at the Medical University of Vienna. **Methods**: In this retrospective study, 352 children who underwent ROP screening between 2018 and 2021 with a gestational age (GA) ≤ 32 weeks and/or a birth weight (BW) ≤ 1500 g were included. **Results**: ROP was found in 144 (40.9%) of the 352 screened premature infants, with 17 (4.8%) requiring treatment. Significant risk factors included GA and BW, while sex and pregnancy type were not significant. The mean GA was 27.7 ± 2.5 weeks, and the mean BW was 989.1 ± 359.7 g. Infants with ROP had a lower GA (25.9 ± 1.7 weeks) and BW (778.6 ± 262.4 g) than those without ROP (28.9 ± 2.2 weeks; 1134.9 ± 345.9 g). GA and BW were significantly lower in infants developing ROP (*p* < 0.001). Stage 2 ROP was the most common severity in 74 children (51.4%). Laser therapy was the most common first-line treatment, used in 11 infants (64.7%), followed by anti-VEGF therapy, used in 6 infants (35.3%). Children were treated within 1.0 ± 0.6 days on average. Of the 17 infants treated, 14 (82.4%) showed initial regression. Three infants (17.6%) required re-treatment: two with initial anti-VEGF therapy and one after laser therapy. **Conclusions**: The findings provide insights into ROP’s prevalence and treatment preferences at a university tertiary care center. GA and BW were confirmed to be significant predictors, aiding in early detection and informing treatment decisions. These insights will enable comparisons with similar studies and contribute to improved patient care.

## 1. Introduction

Retinopathy of prematurity (ROP) is a multifactorial retinal disease characterized by proliferative vasculopathy [1,2]. The development of ROP is divided into two phases, where an initially suppressed maturation of the vascular system due to premature birth is followed by increased vascular growth [3]. There are five stages of ROP: In stage 1, a thin demarcation line separates the avascular retina from the vascularized retina. In stage 2, a ridge arises from the demarcation line. Stage 3 is characterized by extraretinal fibrovascular proliferation. Stage 4 involves partial retinal detachment, and stage 5 is marked by total retinal detachment.

Globally, ROP is a leading cause of potential vision impairment in children and remains a major cause of childhood blindness [4,5]. It affects preterm infants (born before week 37), particularly those with low BW (<1500 g) and low GA (<32 weeks) [6,7]. Prematurity itself is considered the most significant risk factor for the occurrence of ROP [1]. Other clinical risk factors include low GA, low BW, a high demand for postnatal oxygen supply, multiple births, and male gender. In addition to the amount of oxygen administered, the duration of oxygen supply and strong fluctuations in oxygenation are responsible for the progression of the disease [8,9].

Due to the increasing number and survival rate of extremely preterm infants in recent decades, ROP remains an important threat, especially for this vulnerable group, with the numbers of severe ROP also increasing [10,11]. Although annual fluctuations are observed, there is an overall increasing trend in the incidence of ROP [11]. Recognizing the potential danger of this disease, significant efforts have been made to understand its development, leading to advancements in prevention and treatment strategies [5]. Advances in neonatal care during the 1970s significantly improved the survival rates of extremely preterm infants. However, this success has also led to an increase in the number of children at the highest risk for developing ROP [7,10]. This was followed by a second epidemic, with a greater incidence of ROP [12] and blindness due to ROP in industrialized countries such as the US and Western Europe [13,14,15]. Since the 1990s, a third epidemic of visual impairment due to ROP has occurred specifically in middle-income countries (MICs), primarily attributable to the development of neonatal care but also caused by unregulated oxygen supply as well as insufficient ROP screening and treatment options [13,16].

To prevent permanent visual impairment and blindness, ophthalmological screening for preterm infants with the aforementioned risk factors is essential [17]. The early detection of threatening ROP stages through screening, followed by timely therapeutic intervention, can halt the progression of the disease. The consistent implementation and continuous improvement of ROP screening and treatment options can reduce the occurrence of adverse outcomes and enable affected individuals to achieve good visual development [18]. Current recommendations advocate for targeted therapy involving either laser or anti-VEGF therapy based on the disease stage and affected zone.

## 2. Materials and Methods

### 2.1. Study Design

This retrospective data analysis included premature infants who were examined for ROP between January 2018 and January 2021 at the Medical University of Vienna as part of ROP screening and treated if necessary.

### 2.2. Clinical Assessment

All the data originated from manually documented findings and, if necessary, from the AKIM (AKH Information Management) of the University Clinic for Ophthalmology and Optometry in Vienna. They included premature infants who received an ophthalmological examination as part of the ROP screening in the period from January 2018 to January 2021. The number of cases was defined by the period included in the data analysis.

This study collected data from male and female patients who underwent an eye examination as part of the ROP screening for premature infants at the General Hospital in Vienna. Premature infants with a gestational age ≤ 32 weeks of pregnancy and/or a birth weight ≤ 1500 g were included.

The findings for the right and left eyes were collected for each patient. Among the 704 eyes of the 352 patients, one from each patient was included in the statistical analysis. If both eyes were affected by ROP, the patient was categorized based on the more severely affected eye (higher ROP grade). For patients who developed unilateral ROP, the affected eye was therefore included in the analyses, and these patients were evaluated accordingly as ROP cases and, if applicable, as treatment cases. The therapeutic approach was determined based on which treatment method was administered to patients with the most severe form of ROP. If a patient received different therapeutic interventions at various points during their stay, the patient was categorized according to the therapy method used to treat their ROP at the point of highest severity.

### 2.3. Statistical Analysis

The results of the analysis are presented as the numbers of cases, percentages, means (Ms), standard deviations (SDs), medians (MDs), interquartile ranges (IQRs), and maximum and minimum values. Qualitative variables are reported as absolute and relative frequencies. In order to determine significant differences in continuous variables when comparing two groups, *t*-tests for independent samples were used for normally distributed variables. If the data were not normally distributed, significant differences were determined using the Mann–Whitney U test. The Kolmogorov–Smirnov test was used in advance to test the normality of the distributions of these variables within the sample. For categorical variables, the frequency is specified in absolute and relative values. Chi-square tests were used to compare the two groups.

Several independent groups were compared for normally distributed variables using a single-factor analysis of variance and for non-normally distributed variables using the Kruskal–Wallis test. When using the Kruskal–Wallis test, additional pairwise comparisons were made using the Dunn–Bonferroni post hoc test. A binary logistic regression was used to determine whether birth weight, gestational age, gender, and pregnancy type influenced the probability of developing ROP. To accomplish this, each of the four predictors was first tested individually in the regression model for its predictive power. Subsequently, all four factors were analyzed together in the binary logistic regression with the aim of investigating which predictors retained their predictive power when the other predictors were also present in the model. To avoid the overrepresentation of dependent cases, only one child from each multiple birth was randomly included. Whether birth weight and gestational age were related to the severity of ROP was analyzed using Spearman’s rank correlation. Statistical analyses were conducted with SPSS (IBM Statistics, version 27) for Windows. *p*-values < 0.05 were considered statistically significant.

## 3. Results

In total, 352 infants who underwent screening for ROP were enrolled: 151 (42.9%) female and 201 (57.1%) male infants. The population included 232 (65.9%) single births, 103 twins (29.3%), and 17 triplets (4.8%). Of the 352 included infants, 144 developed retinopathy of prematurity, leading to an ROP incidence of 40.9% between January 2018 and January 2021. Five (1.4%) infants developed unilateral ROP, while four (1.1%) developed left-sided ROP. Moreover, 17 (4.8%) infants developed severe ROP requiring treatment. The characteristics of the children, separated by patients with ROP and those without ROP, are shown in Table 1.

During the observation period, 74 children (51.4%) developed stage 1 ROP, 53 (36.8%) developed stage 2, 16 (11.1%) developed stage 3, and 1 (0.7%) developed stage 4. No stage 5 ROP was diagnosed. Zone II was the most affected, with 131 children (91.0%) developing the disease in this zone. Zone I was affected in 10 children (6.9%), and Zone III in 3 children (2.1%). Additional ophthalmological findings included plus disease in 65 cases (45%), retinal or vitreous hemorrhages in 33 patients (23%), vitreous opacities in 15 patients (10%), and present tunica vasculosa lentis in 11 patients (8%).

The mean gestational age of the entire study population was 27.7 ± 2.5 weeks, and the mean birth weight was 989.1 ± 359.7 g. The median gestational age was 28 weeks (IQR = 3), and the median birth weight was 927.5 g (IQR = 500).

Differences in the mean gestational age between infants with and without ROP (25.9 ± 1.7/28.9 ± 2.2) and in the mean birthweight (778.6 ± 262.4/1134.9 ± 345.9) were observed, and a significantly lower median gestational age and median birth weight in infants with ROP were confirmed by Mann–Whitney U tests (median = 26 weeks, IQR = 3; median = 29 weeks, IQR = 4; *p* < 0.001).

Both a lower gestational age and lower birth weight correlated with higher ROP severity (rs = −0.339, *p* < 0.001; rs = −0.334, *p* < 0.001). ROP developed in 75 males (37.3%) and 69 females (45.7%). There was no significant difference in ROP incidence between genders (chi-square (1) = 2.506, *p* = 0.113).

In the ROP group, 104 (72.2%) were singletons, 37 (25.7%) twins, and 3 (2.1%) triplets. Among the preterm infants without ROP, 128 (61.5%) were singletons, 66 (31%) twins, and 14 (6.7%) triplets. Singletons comprised the majority of the sample, with 44.8% developing ROP, compared to 35.9% of the twins and 17.6% of the triplets. In a single-factor analysis of variance using the Kruskal–Wallis test, it was investigated whether the pregnancy types differed in terms of gestational age and birth weight in order to determine whether these factors were possible confounding variables. Gestational age and birth weight differed significantly across pregnancy types (Kruskal–Wallis-H (2) = 32.32, *p* < 0.005), with singletons showing a higher birth weight than multiples.

### 3.1. Correlations

**Figure 1:**
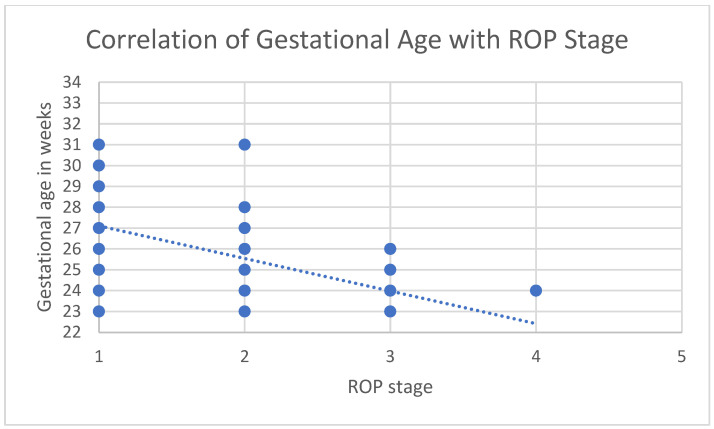
Correlation of gestational age (weeks) with stage of ROP (1–5).

**Figure 2:**
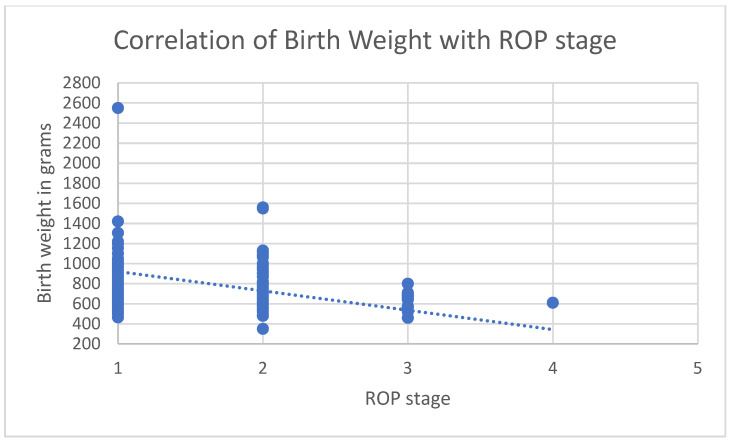
Correlation of birth weight (grams) with stage of ROP (1–5).

A lower gestational age was significantly associated with a higher risk of developing ROP (chi-square (1) = 121.578, *p* < 0.001). Specifically, with each additional week of gestation, the relative probability of developing ROP decreased by 53.7% (OR = 0.463, 95% CI [0.385, 0.556]). Similarly, birth weight was identified as a significant predictor for ROP (chi-square (1) = 82.971, *p* < 0.001). A 1-g increase in birth weight reduces the relative probability of developing ROP by 0.4% (OR = 0.996, 95% CI [0.995, 0.997]). In contrast, neither sex (chi-square (1) = 2.651, *p* = 0.103) nor type of pregnancy (chi-square (1) = 2.260, *p* = 0.232) were significant risk factors. The correlation of gestational age with the severity of ROP and birth weight with the severity of ROP is presented in Figure 1 and Figure 2.

Subsequently, a binary logistic regression model that included all four risk factors was analyzed to understand their combined effect on ROP risk. This comprehensive analysis was crucial, as all four characteristics typically present simultaneously in a clinical context, contributing to the risk profiles of individual patients. Combined regression analysis indicated gestational age as the most significant predictor. Each additional week of gestation continued to be associated with a 50.3% reduction in the relative probability of developing ROP (OR = 0.497, 95% CI [0.397, 0.621]).

### 3.2. Treatment of ROP

In total, 11.8% (n = 17) of the ROP cases required treatment, all with stage 3 ROP. Therefore, ROP requiring treatment occurred with an incidence of 4.8%. Among the 17 children receiving treatment, zone I was affected in 4 (23.5%), while zone II was affected in 13 (76.5%). No AP-ROP was diagnosed during the observation period.

Laser photocoagulation was the primary treatment in 64.7% (11 patients, 21 eyes), while 35.3% (6 patients, 12 eyes) received anti-VEGF injections as the first treatment. The average time between the diagnosis of ROP in its most severe form and treatment was 1 day (0.71 ± 0.6). Treatment was provided either on the same day or no more than 2 days after diagnosis.

Following the approval of ranibizumab for ROP treatment, our analysis investigated whether there was a change in therapy applications over the years. In 2018, six (75%) primary laser coagulations and two (25%) anti-VEGF therapies were performed. In 2019, all four (100%) patients treated received laser therapy. By 2020, this had decreased to one (33.3%) primary laser coagulation and two (66.7%) anti-VEGF therapies, and in January 2021, there were no primary laser coagulations and two (100%) anti-VEGF therapies. The therapeutic method was chosen based on the individual circumstances of the patients and after careful consideration of the risk factors. It was considered that anti-VEGF therapy, particularly for ROP in zone I, is superior to laser treatment, as it allows for postoperative vascularization (24). However, this form of treatment must be avoided in cases of recent ocular inflammation to prevent endophthalmitis. For ROP involving zone II, laser coagulation therapy was preferred, provided the patients were suitable candidates for the anesthesia required for this treatment.

Of the 17 children receiving treatment, 14 (82.4%) showed successful regression of ROP. Three children (17.6%) required at least one re-treatment. This impressive success rate underscores the efficacy of the treatment modalities employed and highlights the importance of timely and appropriate intervention.

Laser coagulation and anti-VEGF therapy were used as both an initial treatment and possible follow-up treatment. Vitrectomy with cerclage was used in one patient and only as part of a re-treatment after an insufficient response to the initial therapy. This explains the fact that the total number of treatments using a particular method exceeds the total number of patients with ROP requiring treatment. In total, 23 treatments were performed, including 13 laser treatments (56.5%), 7 anti-VEGF injections (30.4%), 2 vitrectomies (8.7%), and 1 cerclage (4.3%).

Among the successfully treated patients after the first treatment, 10 (71.4%) children received initial laser therapy, and 4 (28.6%) children were treated with anti-VEGF therapy. It should be noted that three children (17.6%) did not achieve complete regression after the first treatment, which may reflect individual variations in disease progression or responsiveness to the therapy. Regarding the patients who required additional treatment, two had initially received anti-VEGF therapy, and one had initially received laser treatment. The last-mentioned was initially diagnosed with bilateral stage 3 ROP in zone I. Despite the central zone I localization, diode laser therapy in both eyes was indicated due to recently developed conjunctivitis to avoid the risk of endophthalmitis. After 2 months, there was a bilateral progression of the disease, manifesting as significant elevation and traction at the retinal ridge with central involvement. These findings necessitated an additional laser treatment with a central barricade in both eyes. Another 2 months later, bilateral progression to stage 4 ROP was diagnosed, requiring bilateral vitrectomy. While the right eye remained stable thereafter, further intervention was indicated 2 months later for the left eye due to stage 4 ROP with temporal retinal detachment. This involved another vitrectomy and the placement of a scleral buckle in the left eye.

This case illustrates the complex and challenging course of ROP in a premature infant despite early and intensive treatment measures. Such cases underscore the need for continuous monitoring and adaptive therapeutic approaches to prevent disease progression and preserve visual function as much as possible. Notably, all the patients who required additional treatment had very low gestational ages (≤25 weeks) and birth weights (≤610 g). The outcomes of the different treatment methods in stages 3 and 4 for the 17 treated patients are presented in Table 2, categorized by gestational age (≤24, 25 and ≥26 weeks). The last aforementioned patient, who showed no initial treatment success after the first laser therapy for ROP in stage 3, zone I, and whose condition worsened after a second laser treatment, eventually developed stage 4 ROP. Therefore, this patient is classified as stage 4 in the table. The table demonstrates that all the other patients who received laser therapy, regardless of gestational age, showed initial treatment success in the form of ROP regression. Because only three patients required follow-up treatment, these findings cannot be generalized.

## 4. Discussion

In this study, we analyzed ROP screening and treatment procedures at the Medical University of Vienna from January 2018 to January 2021. The results can contribute to enhancing current knowledge of ROP and improving the management of affected patients.

Globally, over the past 15 years, incidence rates have varied between 9% and 41%, depending on the country and study population [19]. The overall incidence of ROP found in our sample was 40.9%, which is similar to that in previous studies. Larsen et al. examined over 800 newborns in Germany between 2010 and 2015, reporting incidences between 23.4% and 32.7% [20], while Busik et al. reported a similar incidence of 33.9% between 2009 and 2019 [11]. Hellström et al. found an incidence of 31.9% in Sweden between 2008 and 2015. Variations in incidence rates within different regions were also noted in Sweden, ranging from 16.7% to 47.5%, depending on regional healthcare measures and clinical management. These differences can be attributed to various neonatal care measures [21].

A treatment-requiring ROP was observed in our cohort with an incidence of 4.8%, consistent with European studies reporting treatment-requiring ROP rates of 2.5% (2012–2016) and 4.1% (2006–2016) in Germany, 5.7% in Sweden (2008–2015), and a very low incidence of 1.2% in Switzerland (2005–2015). Overall, the number of ROP cases requiring treatment in Europe, therefore, appears to be more or less stable [20,21,22,23].

With regard to the manifestation of ROP, it was shown in this study that zone II is by far the most frequently affected zone, being affected in 91% (n = 131) of the patients at the time of maximum manifestation. Other studies also found that zone II ROP accounted for the largest proportion of cases at 86.6% [20]. Walz et al. showed that, over a 5-year period, stage 3+, zone II disease was the most common indication for treatment at 70–90% [24]. The present study also confirmed that central and, therefore, potentially, even more dangerous zone I disease and severe stages 4 and 5 are very rare. Similar to our study, in which stage 5 disease did not occur at all and a stage 4 case was only observed as a progression in one patient after laser therapy in stage 3, Larsen et al. reported no stage 4 in a study cohort of 863 children, and only a single case of stage 5 that occurred after anti-VEGF treatment of a stage 3 case [20].

While stages 4 and 5 have become rare in high- and middle-income countries, many preterm infants in low-income countries continue to develop a severe stage of ROP [6]. A 2019 paper by Bowe et al. provides an overview of the prevalence of ROP in low-income countries. It shows that, in India, between 2010 and 2018, 20% of the children screened were diagnosed with ROP requiring treatment, and in Mexico, between 2011 and 2015, 33% of the 261 patients screened required treatment. Stage 4 occurred in 11% of ROP patients and stage 5 in 13% [25]. The biggest problems are the insufficient awareness and recognition of this relevant eye disease and its long-term complications and the lack of regulation of oxygen supply. A further obstacle is the fact that funding for ROP screening programs and adequate treatment methods is a major challenge for some countries [25].

The choice between laser coagulation and anti-VEGF therapy is crucial for ROP treatment. In this study, laser coagulation was the most frequently applied treatment, with 64.7% (11 out of 17 patients) receiving it as the first treatment and 56.5% (13 out of 23 interventions) overall, especially for zone II diseases. Tiryaki et al. also described laser coagulation as the preferred treatment option in a paper published in 2018 [26]. According to the recommendations that provide for timely therapy [27], our patients in need of therapy were successfully treated after 1 day (0.71 ± 0.6) on average and, in any case, within 48 h.

ROP can progress to a degree of severity that indicates a new treatment after both anti-VEGF therapy and laser coagulation [23]. The question of which treatment method performs better in terms of the number of follow-up treatments remains a central research question. The BEAT-ROP study confirmed the effectiveness of anti-VEGF therapy for zone I diseases in stage 3+, finding that the recurrence rate after bevacizumab treatment was 6%, significantly lower than the 26% rate after laser therapy [27]. Regarding the safety and efficacy of anti-VEGF therapies, different dosages and agents have been investigated. The CARE-ROP study found that reduced doses of ranibizumab (0.2 and 0.12 mg) were effective [28]. Wu et al. [29] showed minimal and the RAINBOW study [30] showed no systemic VEGF suppression with ranibizumab, making it appear to be a safer option than bevacizumab.

In this study, the majority of the patients receiving treatment, 14 out of 17 (82.4%), showed successful regression of the ROP after just one treatment without the need for further therapy. In this study, follow-up treatment after anti-VEGF was necessary in two cases, while only one patient with laser therapy required further treatment. Due to the very small number of patients treated, it is not possible to make a statistically meaningful comparison to determine which treatment method actually requires fewer follow-up treatments. On the other hand, this is a positive finding, as it can be deduced from this that the two first-line therapies, anti-VEGF and laser treatment, show good efficacy.

The recurrence rates after initial treatments varied across studies. While Tiryaki et al. [26] and the BEAT-ROP study [31] found lower recurrence rates after anti-VEGF therapy, Walz et al. (21% vs. 19%) and Hwang et al. (16% vs. 3%) reported higher recurrence rates after anti-VEGF compared to laser treatment [24,32]. Hu et al. found that recurrences with retinal detachment after bevacizumab therapy could occur up to a postmenstrual age of 49 to 69 weeks [33]. The BEAT-ROP study chose 54 weeks postmenstrual as an endpoint, potentially missing late recurrences [34,35]. Follow-up studies confirmed a low number of late recurrences and a lower rate of severe myopia after bevacizumab injection compared to laser treatment [36].

Nevertheless, anti-VEGF injections are gaining importance. Data from Hannover showed that anti-VEGF therapy has accounted for an increasing share of treatments since 2014 [23]. Tiryaki et al. and Akman et al. described anti-VEGF therapy as effective, especially for zone I diseases [23,26]. In our sample, 35.3% of treated children (6 out of 17) received an anti-VEGF injection as the first-line treatment. Four patients required only one injection, while two needed follow-up surgery due to ROP progression. Zone I diseases were mainly treated with anti-VEGF therapy unless there was a recent inflammatory condition. Regarding the approval of ranibizumab in 2019 for the treatment of ROP, it was of interest to investigate whether there had been an increase in the number of anti-VEGF therapies performed since then. Our results indicate that the number of primary laser coagulations decreased from six treatments in 2018 to one in 2020. In January 2021, no laser coagulations but two anti-VEGF injections were performed. While one might expect a significant increase in anti-VEGF therapy, our data showed a nearly stable application of this procedure, with two treatments each in 2018, 2020, and January 2021.

It is noteworthy that therapy decisions were made zone-dependently, with more peripheral findings (zones II and III) observed in 2018, for which laser therapy was preferred. This might have influenced the preference for laser therapy in that year. Additionally, given the generally small number and varying number of cases reaching a treatment-requiring stage each year, direct comparisons are further complicated. In summary, it is difficult to make generalizable statements regarding a specific trend in ROP therapy, as the decision must be individualized considering the specific clinical scenario, the zone of ROP involvement, and the patient’s overall health and suitability for anesthesia. Our therapy decisions were based on current recommendations [27,34].

Laser therapy is advantageous, as it avoids opening the globe, thereby eliminating the risk of endophthalmitis. Typically, one treatment session is sufficient to achieve therapeutic goals. However, this procedure is time-consuming, usually necessitates general anesthesia, and requires significant expertise from the treating physician [34]. Nevertheless, irreversible destruction of the peripheral retina can lead to visual field constrictions or myopia. Anti-VEGF therapy, on the other hand, is characterized by a short treatment duration and can often be performed without general anesthesia, reducing associated risks. This approach allows for postoperative retinal vascularization, potentially preventing visual field loss, and has been associated with a reduction in the development of ROP-related myopia. Despite these advantages, anti-VEGF therapy carries an inherent risk of endophthalmitis due to the intraocular injections. The therapeutic effect may diminish over time, necessitating repeated treatments or subsequent laser therapy if the ROP reactivates. Additionally, late recurrences are possible, necessitating long-term, regular follow-up to monitor and manage potential relapses [34,37].

The central aim of this study was to investigate the risk factors of gestational age, birth weight, gender, and type of pregnancy in relation to the development of ROP. Our findings confirm that particularly low gestational age and birth weight are significant risk factors for ROP.

A low gestational age was strongly associated with an increased risk of developing ROP (chi-square (1) = 121.578, *p* < 0.001). Each additional week of gestation reduces the relative risk of ROP by 50.3% (OR = 0.497, 95% CI [0.397, 0.621]). This highlights the importance of obstetric care for reducing the preterm birth rate. Larsen et al. [20] and Fortes Filho et al. [38] also reported the importance of gestational age as a risk factor, with Larsen et al. finding a median gestational age of 27 weeks and Fortes Filho et al. reporting a 43% increased risk of severe ROP for children born before 28 weeks.

Birth weight was also a significant risk factor, with each additional gram reducing the relative risk of ROP by 0.4% (OR = 0.996, 95% CI 0.995, 0.997). Seiberth and Linderkamp [39] identified birth weight as an independent risk factor, while Lundgren et al. [40] suggested that postnatal growth retardation might play a larger role. This study showed that birth weight, which was a significant factor when considered alone, lost its predictive ability for the risk of developing ROP in the combined model. This might suggest that birth weight becomes irrelevant once gestational age is accounted for. In clinical settings, where both gestational age and birth weight are known, gestational age should be considered the more reliable predictor for the relative probability of developing ROP.

Gender was not identified as a significant risk factor in this study. Despite lower IGF-1 levels in male fetuses [41,42], our study showed neither a significant difference in group comparison nor an impact on ROP risk in binary logistic regression. Similar results were reported by Hakeem et al. [43] and Feghi et al. [44], while Darlow et al. [45] found an association between male gender and ROP.

It is known that the risk of premature birth and low gestational age is increased in multiple pregnancies, and consequently, an increased incidence of ROP would be expected [46]. In our patients, multiple births had no significant impact on the risk of developing ROP, as also reported by Blumenfeld et al. [47]. Friling et al. [48] found that, in twin births, the lower birth weight of the second-born twin explained the higher risk. Nevertheless, Li et al. [49] found a higher ROP incidence in twins than in singletons, and Motta et al. [50] demonstrated a higher incidence of any stage of ROP except threshold-ROP in twins and triplets. Furthermore, additional complications that affect multiple births and have a potential impact on the development of ROP should also be considered. According to previous studies, twins affected by twin-to-twin transfusion syndrome during pregnancy showed an increased risk of developing ROP, presumably because they are exposed to hypoxic conditions that affect the development of the retina [51,52]. However, regarding our findings, predictions about the probability of developing ROP cannot be made based on sex or type of pregnancy. As our study had the limitation of a smaller number of infants born from multiple pregnancies, especially triplets, compared to singletons, the absence of association may have been due to that.

Because prematurity is significantly associated with the occurrence of ROP, as this study shows, this ocular disease will continue to occur as long as preterm births happen. Additionally, preterm infants often require high oxygen supplementation for survival, which further increases the risk of developing ROP [53].

Current studies are investigating the possibility of preventing ROP through the identification of biomarkers associated with its pathogenesis [54]. High levels of several oxidative stress markers, which are indicative of oxidative stress levels in preterm infants, are suspected to be linked to increased susceptibility to ROP. However, there is no conclusive proof that measuring and reducing oxidative stress alone will prevent retinal changes and ROP [54]. Regarding the prevention of ROP, further research is needed to validate whether targeted antioxidant therapies or careful oxygen management, guided by oxidative stress marker levels, could effectively prevent or reduce the severity of ROP in clinical settings.

## 5. Conclusions

Our study found that gestational age and birth weight are the most important risk factors for the development of ROP. Higher gestational age can significantly reduce the risk of developing ROP. The findings from this study underscore that the early and continuous screening of disease progression and timely adjustment of targeted interventions by experienced ophthalmologists and neonatologists enhance the likelihood of successful ROP regression.

We showed that both laser therapy and anti-VEGF therapy result in successful ROP regression. However, the question of which treatment is superior regarding the need for follow-up remains unanswered. Ongoing research and refinement of treatment strategies remain essential to further improve long-term outcomes and address the specific requirements of patients.

Whether it will be possible to improve ROP prevention by assessing risk using biomarkers alongside traditional factors like birth weight and gestational age remains an intriguing area of study.

## Figures and Tables

**Table 1:** Comparison of characteristics of enrolled patients with and without ROP (n = 352).

	ROP ^1^ N (%) or Median (IQR) ^2^	NON-ROP N (%) or Median (IQR) ^2^	*p*-Value
Birth weight (grams) Gestational age (weeks)	711 (326.3)	1125 (427.5)	*p* < 0.001
	26 (3)	29 (4)	*p* < 0.001
**Gender** Female Male	69 (47.9%)	82 (39.4%)	*p* = 0.113
	75 (52.1%)	126 (60.6%)	
**Pregnancy type** Singleton birth Twin Triplet			*p* = 0.042
	104 (72.2%)	128 (61.5%)	
	37 (25.7%)	66 (31.7%)	
	3 (2.1%)		
**Total**	144 (100%)	208 (100%)	

^1^ ROP = retinopathy of the prematurity; ^2^ IQR = interquartile range.

**Table 2:** Treatment outcomes based on ROP stage and gestational age. N = 17 treated patients.

ROP Stage	Gestational Age (Weeks)	Treated Patients (N = 17)	Type of Treatment	Initial Success ^1^ N (%)	Type of Follow-Up Treatment
Stage 3 (N = 16)	≤24	8	Laser (N = 5) Anti-VEGF (N = 3)	5 (100%) 2 (66.7%)	Laser (N = 1)
	25 ≥26	5 3	Laser (N = 2) Anti-VEGF (N = 3) Laser (N = 3) Anti-VEGF (N = 0)	2 (100%) 2 (66.7%) 3 (100%)	Anti-VEGF (N = 1) -
Stage 4 (N = 1)	≤24 25 ≥26	1 0 0	Vitrectomy (N = 1) - -	0 (0%) - -	Vitrectomy and cerclage (N = 1) - -

^1^ Initial success: patients who showed regression of ROP without requiring a secondary treatment.

## Data Availability

The data presented in this study are not publicly available due to privacy concerns and ethical restrictions. Access to the data can be requested from the corresponding author, subject to approval by the Ethics Committee of the Medical University of Vienna (protocol code EK-Nr. 2148).

## References

[1] Esser J., Gareis O., Lang G.E., Recker D., Spraul C.W., Wagner P., Lang G.K. (2019). Augenheilkunde.

[2] Stahl A., Lagrèze W.A., Agostini H.T. (2012). Pathogenese der Frühgeborenenretinopathie. Der Ophthalmol..

[3] Smith L.E.H. (2003). Pathogenesis of retinopathy of prematurity. Seminars in Neonatology.

[4] Kong L., Fry M., Al-Samarraie M., Gilbert C., Steinkuller P.G. (2012). An update on progress and the changing epidemiology of causes of childhood blindness worldwide. J. Am. Assoc. Pediatr. Ophthalmol. Strabismus JAAPOS.

[5] Wood E.H., Chang E.Y., Beck K., Hadfield B.R., Quinn A.R., Harper C.A. (2021). 80 Years of vision: Preventing blindness from retinopathy of prematurity. J. Perinatol..

[6] Sabri K., Ells A.L., Lee E.Y., Dutta S., Vinekar A. (2022). Retinopathy of Prematurity: A Global Perspective and Recent Developments. Pediatrics.

[7] Lang G.E., Lang G.K., Esser J. (2015). Schlaglicht Augenheilkunde: Kinderophthalmologie.

[8] Hübler A., Jorch G., Arenz S., Avenarius S., Bachmaier N., Berger A., Bittrich H.-J., Brockmann P.E., Brune T., Bührer C. (2019). Neonatologie [Internet].

[9] Gortner L., Meyer S. (2018). Duale Reihe Pädiatrie [Internet].

[10] Ancel P.Y., Goffinet F., Kuhn P., Langer B., Matis J., Hernandorena X., Chabanier P., Joly-Pedespan L., Lecomte B., EPIPAGE-2 Writing Group (2015). Survival and morbidity of preterm children born at 22 through 34 weeks’ gestation in France in 2011: Results of the EPIPAGE-2 cohort study. JAMA Pediatr..

[11] Busik V., Lorenz B., Mais C., Jäger M., Friedburg C., Andrassi-Darida M., Ehrhardt H., Hubert M. (2023). 10 Jahre Screening auf Frühgeborenenretinopathie (2009–2019). Die Ophthalmol..

[12] Valentine P.H., Jackson J.C., Kalina R.E., Woodrum D.E. (1989). Increased survival of low birth weight infants: Impact on the incidence of retinopathy of prematurity. Pediatrics.

[13] Gilbert C., Malik A.N.J., Nahar N., Das S.K., Visser L., Sitati S., Ademola-Popoola D.S. (2019). Epidemiology of ROP update—Africa is the new frontier. Seminars in Perinatology.

[14] Bhatnagar A., Skrehot H.C., Bhatt A., Herce H., Weng C.Y. (2023). Epidemiology of Retinopathy of Prematurity in the US From 2003 to 2019. JAMA Ophthalmol..

[15] Ahmed I., Hoyek S., Patel N.A. (2023). Global Disparities in Retinopathy of Prematurity: A Literature Review. Seminars in Ophthalmology.

[16] Gilbert C., Rahi J., Eckstein M., O’Sullivan J., Foster A. (1997). Retinopathy of prematurity in middle-income countries. Lancet.

[17] Stahl A., Göpel W. (2015). Screening and Treatment in Retinopathy of Prematurity. Dtsch. Arztebl. Int..

[18] Li L., Gao Y., Chen W., Han M. (2022). Screening for retinopathy of prematurity in North China. BMC Ophthalmol..

[19] Hong E.H., Shin Y.U., Cho H. (2021). Retinopathy of prematurity: A review of epidemiology and current treatment strategies. Clin. Exp. Pediatr..

[20] Larsen P.P., Bründer M.C., Petrak M., Jehle V., Lagrèze W.A., Holz F.G., Stahl A., Krohne T.U. (2018). Frühgeborenenretinopathie-Screening: Trends über die vergangenen 5 Jahre an zwei deutschen Universitätskliniken. Ophthalmologe.

[21] Holmström G., Tornqvist K., Al-Hawasi A., Nilsson Å., Wallin A., Hellström A. (2018). Increased frequency of retinopathy of prematurity over the last decade and significant regional differences. Acta Ophthalmol..

[22] Gerull R., Brauer V., Bassler D., Laubscher B., Pfister R.E., Nelle M., Müller B., Gerth-Kahlert C., Adams M. (2018). Incidence of retinopathy of prematurity (ROP) and ROP treatment in Switzerland 2006-2015: A population-based analysis. Arch. Dis. Child.-Fetal Neonatal Ed..

[23] Akman S.H., Pfeil J.M., Stahl A., Ehlers S., Böhne C., Bohnhorst B., Framme C., Brockmann D., Bajor A., Jacobsen C. (2022). Epidemiologie und Therapie der behandlungsbedürftigen Frühgeborenenretinopathie. Die Hannoveraner Daten im Retina.net ROP-Register von 2001 bis 2017. Der Ophthalmol..

[24] Walz J.M., Rop-Register-Studiengruppe R., Bemme S., Reichl S., Akman S., Breuß H., Süsskind D., Glitz B., Müller V.C., Wagenfeld L. (2018). Behandelte Frühgeborenenretinopathie in Deutschland. Ophthalmologe.

[25] Bowe T., Nyamai L., Ademola-Popoola D., Amphornphruet A., Anzures R., Cernichiaro-Espinosa L.A., Duke R., Duran F., Martinez-Castellanos M.A., Multani D.P.K. (2019). The current state of retinopathy of prematurity in India, Kenya, Mexico, Nigeria, Philippines, Romania, Thailand, and Venezuela. Digit. J. Ophthalmol. DJO.

[26] Tiryaki Demir S. (2019). Evaluation of Treatment Models in the Treatment of Retinopathy of Prematurity. SiSli Etfal Hastan Tip Bul. Med. Bull. Sisli Hosp..

[27] Maier R.F., Hummler H., Kellner U., Krohne T.U., Lawrenz B., Lorenz B., Mitschdörfer B., Roll C., Stahl A. (2021). Augenärztliche Screening-Untersuchung bei Frühgeborenen. Ophthalmol. Z. Dtsch. Ophthalmol. Ges..

[28] Stahl A., Krohne T.U., Eter N., Oberacher-Velten I., Guthoff R., Meltendorf S., Ehrt O., Aisenbrey S., Roider J., Gerding H. (2018). Comparing Alternative Ranibizumab Dosages for Safety and Efficacy in Retinopathy of Prematurity. JAMA Pediatr..

[29] Wu W.C., Shih C.P., Lien R., Wang N.K., Chen Y.P., Chao A.N., Chen K.-J., Chen T.-L., Hwang Y.-S., Lai C.-C. (2017). Serum vascular endothelial growth factor after Bevacizumab or Ranibizumab treatment for retinopathy of prematurity. Retina.

[30] Stahl A., Lepore D., Fielder A., Fleck B., Reynolds J.D., Chiang M.F., Li J., Liew M., Maier R., Zhu Q. (2019). Ranibizumab versus laser therapy for the treatment of very low birthweight infants with retinopathy of prematurity (RAINBOW): An open-label randomised controlled trial. Lancet.

[31] Mintz-Hittner H.A., Kennedy K.A., Chuang A.Z., BEAT-ROP Cooperative Group (2011). Efficacy of intravitreal bevacizumab for stage 3+ retinopathy of prematurity. N. Engl. J. Med..

[32] Hwang C.K., Hubbard G.B., Hutchinson A.K., Lambert S.R. (2015). Outcomes after Intravitreal Bevacizumab versus Laser Photocoagulation for Retinopathy of Prematurity: A 5-Year Retrospective Analysis. Ophthalmology.

[33] Hu J., Blair M.P., Shapiro M.J., Lichtenstein S.J., Galasso J.M., Kapur R. (2012). Reactivation of Retinopathy of Prematurity After Bevacizumab Injection. Arch. Ophthalmol..

[34] Deutsche Ophthalmologische Gesellschaft e. V. (DOG), Retinologische Gesellschaft e. V. (RG), Berufsverband der Augenärzte Deutschlands e. V. (BVA) (2020). Stellungnahme der Deutschen Ophthalmologischen Gesellschaft, der Retinologischen Gesellschaft und des Berufsverbands der Augenärzte Deutschlands zur Anti-VEGF-Therapie der Frühgeborenenretinopathie. Klin. Monatsblätter Augenheilkd..

[35] Stahl A. (2018). Studienüberblick zur Frühgeborenenretinopathie. Der Ophthalmologe.

[36] Geloneck M.M., Chuang A.Z., Clark W.L., Hunt M.G., Norman A.A., Packwood E.A., Tawansy K.A., Mintz-Hittner H.A., for the BEAT-ROP Cooperative Group (2014). Refractive Outcomes Following Bevacizumab Monotherapy Compared With Conventional Laser Treatment: A Randomized Clinical Trial. JAMA Ophthalmol..

[37] Marlow N., Stahl A., Lepore D., Fielder A., Reynolds J.D., Zhu Q., Weisberger A., Stiehl D.P., Fleck B. (2021). 2-year outcomes of ranibizumab versus laser therapy for the treatment of very low birthweight infants with retinopathy of prematurity (RAINBOW extension study): Prospective follow-up of an open label, randomised controlled trial. Lancet Child Adolesc. Health.

[38] Fortes Filho J.B., Eckert G.U., Valiatti F.B., dos Santos P.G.B., da Costa M.C., Procianoy R.S. (2010). The influence of gestational age on the dynamic behavior of other risk factors associated with retinopathy of prematurity (ROP). Graefe’s Arch. Clin. Exp. Ophthalmol..

[39] Seiberth V., Linderkamp O. (2000). Risk Factors in Retinopathy of Prematurity: A Multivariate Statistical Analysis. Ophthalmologica.

[40] Lundgren P., Kistner A., Andersson E.M., Hansen Pupp I., Holmström G., Ley D., Niklasson A., Smith L.E.H., Wu C., Hellström A. (2014). Low Birth Weight Is a Risk Factor for Severe Retinopathy of Prematurity Depending on Gestational Age. PLoS ONE.

[41] Engström E., Niklasson A., Wikland K.A., Ewald U., Hellström A. (2005). The Role of Maternal Factors, Postnatal Nutrition, Weight Gain, and Gender in Regulation of Serum IGF-I among Preterm Infants. Pediatr. Res..

[42] Enninga E.A.L., Nevala W.K., Creedon D.J., Markovic S.N., Holtan S.G. (2015). Fetal Sex-Based Differences in Maternal Hormones, Angiogenic Factors, and Immune Mediators During Pregnancy and the Postpartum Period. Am. J. Reprod. Immunol..

[43] Hakeem A.H.A.A., Mohamed G.B., Othman M.F. (2012). Retinopathy of Prematurity: A Study of Prevalence and Risk Factors. Middle East Afr. J. Ophthalmol..

[44] Feghhi M., Altayeb S.M.H., Haghi F., Kasiri A., Farahi F., Dehdashtyan M., Movasaghi M., Rahim F. (2012). Incidence of Retinopathy of Prematurity and Risk Factors in the South-Western Region of Iran. Middle East Afr. J. Ophthalmol..

[45] Darlow B.A., Hutchinson J.L., Henderson-Smart D.J., Donoghue D.A., Simpson J.M., Evans N.J., Australian and New Zealand Neonatal Network (2005). Prenatal risk factors for severe retinopathy of prematurity among very preterm infants of the Australian and New Zealand Neonatal Network. Pediatrics.

[46] Blondel B., Kogan M.D., Alexander G.R., Dattani N., Kramer M.S., Macfarlane A., Wen S.W. (2002). The Impact of the Increasing Number of Multiple Births on the Rates of Preterm Birth and Low Birthweight: An International Study. Am. J. Public Health.

[47] Blumenfeld L.C., Siatkowski R.M., Johnson R.A., Feuer W.J., Flynn J.T. (1998). Retinopathy of prematurity in multiple-gestation pregnancies. Am. J. Ophthalmol..

[48] Friling R., Rosen S.D., Monos T., Karplus M., Yassur Y. (1997). Retinopathy of prematurity in multiple-gestation, very low birth weight infants. J. Pediatr. Ophthalmol. Strabismus.

[49] Li W.L., He L., Liu X.H., Wang Y.M., Liu J.Q. (2011). Analysis of risk factors for retinopathy of prematurity. Int. J. Ophthalmol..

[50] Motta M., Filho J., Coblentz J., Fiorot C. (2011). Multiple pregnancies and its relationship with the development of retinopathy of prematurity (ROP). Clin. Ophthalmol..

[51] Gschließer A., Stifter E., Neumayer T., Moser E., Papp A., Dorner G., Schmidt-Erfurth U. (2015). Twin-twin transfusion syndrome as a possible risk factor for the development of retinopathy of prematurity. Graefe’s Arch. Clin. Exp. Ophthalmol. Albrecht. Graefe’s Arch. Klin. Exp. Ophthalmol..

[52] El Kateb A., Ville Y. (2008). Update on twin-to-twin transfusion syndrome. Best Pract. Res. Clin. Obstet. Gynaecol..

[53] Saugstad O.D., Aune D. (2013). Optimal Oxygenation of Extremely Low Birth Weight Infants: A Meta-Analysis and Systematic Review of the Oxygen Saturation Target Studies. Neonatology.

[54] Graziosi A., Perrotta M., Russo D., Gasparroni G., D’Egidio C., Marinelli B., Di Marzio G., Falconio G., Mastropasqua L., Li Volti G. (2020). Oxidative Stress Markers and the Retinopathy of Prematurity. J. Clin. Med..

